# Application of three-dimensional transesophageal echocardiography in preoperative evaluation of transcatheter aortic valve replacement

**DOI:** 10.1186/s12872-021-02101-7

**Published:** 2021-06-28

**Authors:** Peng Ding, Chennian Xu, Yang Liu, Xin Meng, Ping Jin, Jiayou Tang, Lanlan Li, Yanyan Ma, Jian Yang

**Affiliations:** 1 Department of Cardiovascular Surgery, Xijing Hospital, Air Force Medical University, 127. Changle West Rd, Xi’an, 710032 China; 2 Department of Ultrasound Medicine, Xijing Hospital, Air Force Medical University, Xi’an, China

**Keywords:** Transcatheter aortic valve replacement, Aortic root sizing, Computed tomography, Three-dimensional transesophageal echocardiography, Aortic valve calcification

## Abstract

**Background:**

Our goal was to determine the accuracy of 3-dimensional transesophageal echocardiography (3D-TEE) compared with that of computed tomography (CT) in the preoperative evaluation for transcatheter aortic valve replacement (TAVR) when the errors caused by inconsistent software and method have been eliminated and the representativeness of the sample has been improved. We also investigated the influence of aortic root calcification on the accuracy of 3D-TEE in aortic annulus evaluations.

**Methods:**

Part I: 45 of 233 patients who underwent TAVR in the department of cardiovascular surgery at the Xijing hospital from January 2016 to August 2019 were studied retrospectively. Materialise Mimics software and the multiplanar reconstruction method were used for evaluation, based on 3D-TEE and CT. The annulus area-derived diameter, the annulus perimeter-derived diameter (Dp), the annulus mean diameter, the left ventricular outflow tract Dp diameter, the sinotubular junction (STJ) diameter-Dp, and the aortic sinus diameter were compared and analyzed. Part II: 31 of 233 patients whose 3D-TEE and CT data were well preserved and in the required format were included. HU450 and HU850 were used as indicators to measure the severity of calcification. The Spearman rank correlation and Linear regression were used to analyze the correlation between aortic root calcification and the accuracy of 3D-TEE in aortic annulus measurement.

**Results:**

The measurement results based on 3D-TEE were significantly lower than those obtained using CT (*P* < 0.05), except for the STJ diameter-Dp in diastole (*P* = 0.11). The correlation coefficient of the two groups was 0.699–0.954 (*P* < 0.01), which also indicated a significant correlation between the two groups. A Bland–Altman plot showed that the ordinate values were mostly within the 95% consistency limit; the consistency of the two groups was good. By establishing the linear regression equation, the two groups can be inferred from each other. The Spearman rank correlation analysis and the Linear regression analysis showed that the influence of aortic calcification on the accuracy of the 3D-TEE annulus evaluation was limited.

**Conclusions:**

Although an evaluation based on 3D-TEE underestimated the results, we can deduce CT results from 3D-TEE because the two methods exhibit considerable correlation and consistency.

***Trial registration*:**

Name: Surgery and Transcatheter Intervention for Structural Heart Diseases. Number: NCT02917980. URL: https://clinicaltrials.gov/ct2/results?term=NCT02917980.

## Introduction

Transcatheter aortic valve replacement (TAVR) has become the treatment of choice for severe aortic diseases. Its safety and efficacy have been widely demonstrated in patients who are inoperable or at medium to high risk for surgery [1, 2]. The US Food and Drug Administration has recently approved the use of the self-expanding valve Evolut R and Evolut PRO systems (Medtronic Inc., Minneapolis, MN, USA) and the balloon-expandable valve Sapien 3 (Edwards Lifesciences Inc., Irvine, CA, USA) for the treatment of severe aortic stenosis (AS) in patients at low risk based on the results of large-sample, multicenter randomized controlled trials [3, 4], which is a milestone for the development of this technology.

Because direct visualization of the aortic root is not possible during the TAVR procedure, accurate preoperative evaluation of the aortic root is of critical importance to reduce the occurrence of complications such as paravalvular aortic regurgitation (AR), valve embolization, coronary obstruction, atrioventricular block, and annulus rupture.

**Fig. 1:**
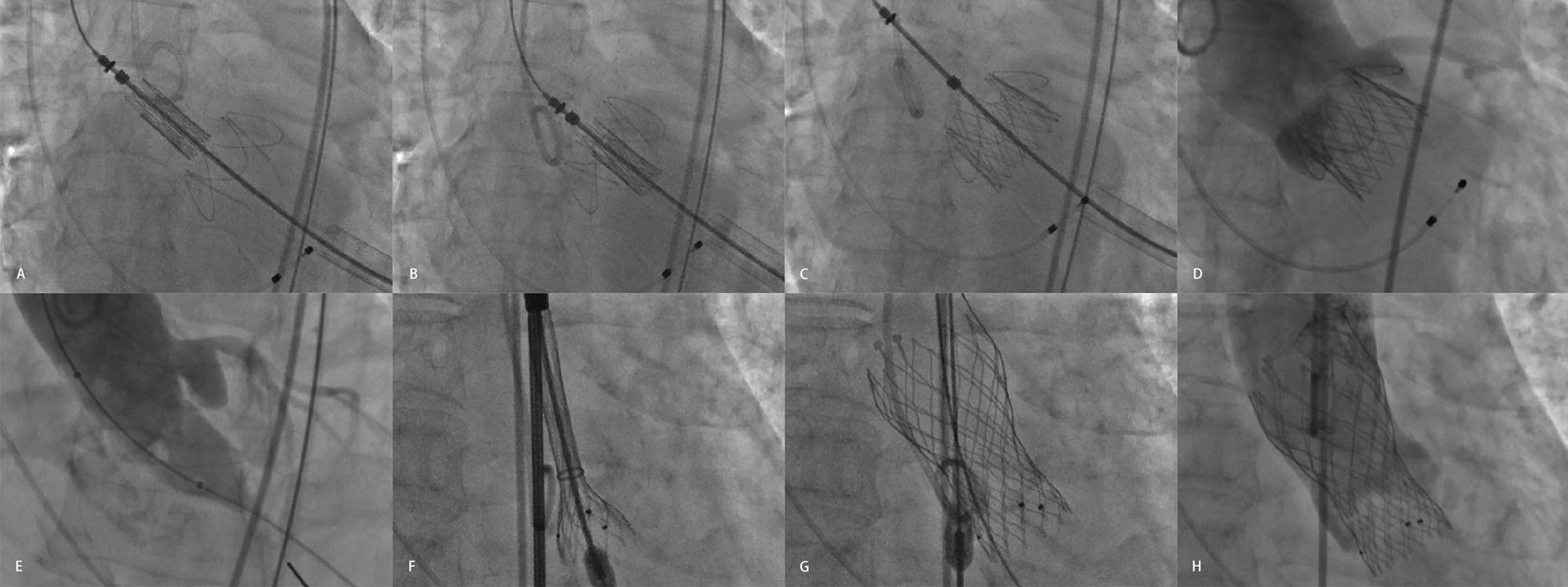
Measurements of the aortic root. **A** Annulus based on computed tomography (CT) measurements. **B** Left ventricular outflow tract (LVOT) based on CT measurements. **C** Sinotubular junction (STJ) based on CT measurements. **D** Sinus of Valsalva (SOV) based on CT scan. **E** Annulus based on 3-dimensional transesophageal echocardiography (3D-TEE). (F) LVOT based on 3D-TEE. **G** STJ based on 3D-TEE. **H** SOV based on 3D-TEE

Computed tomography (CT) is currently the standard of care for preoperative evaluation because it offers more obvious contrast between the vascular and nonvascular structures and facilitates accurate measurements [5] (Fig. 1). However, CT is contraindicated for patients who have renal dysfunction, who are allergic to contrast agents, or who are unsuitable for CT for other reasons.

Three-dimensional transesophageal echocardiography (3D-TEE) has been proposed as an alternative method because it is harmless to humans (Fig. 1). However, accurate 3D-TEE imaging has very strict requirements for respiratory heart rate control and is susceptible to the effects of calcification and other impurities. Currently, there is no standard measurement method based on 3D-TEE. Previous studies showed that measurements based on 3D-TEE tended to underestimate the size of the aortic root [6] and resulted in a higher occurrence of paravalvular regurgitation [7]. However, most of those studies used different software and methods for evaluating CT and 3D-TEE resources, which may contribute to error. In addition, the studies focused mainly on cases with severe AS. Although the indications for TAVR have already been extended to include patients with AR [8–10], there is insufficient evidence regarding the accuracy of 3D-TEE measurements compared with CT using the same software and method for patients scheduled for TAVR, including those with AS and AR.

For the foregoing reasons, the goal of this study was to evaluate the accuracy of 3D-TEE compared with CT in the preoperative measurements of patients with AS and AR using the same software and method and to identify the factors that may influence the results.

## Methods

### Patients

**Fig. 2:**
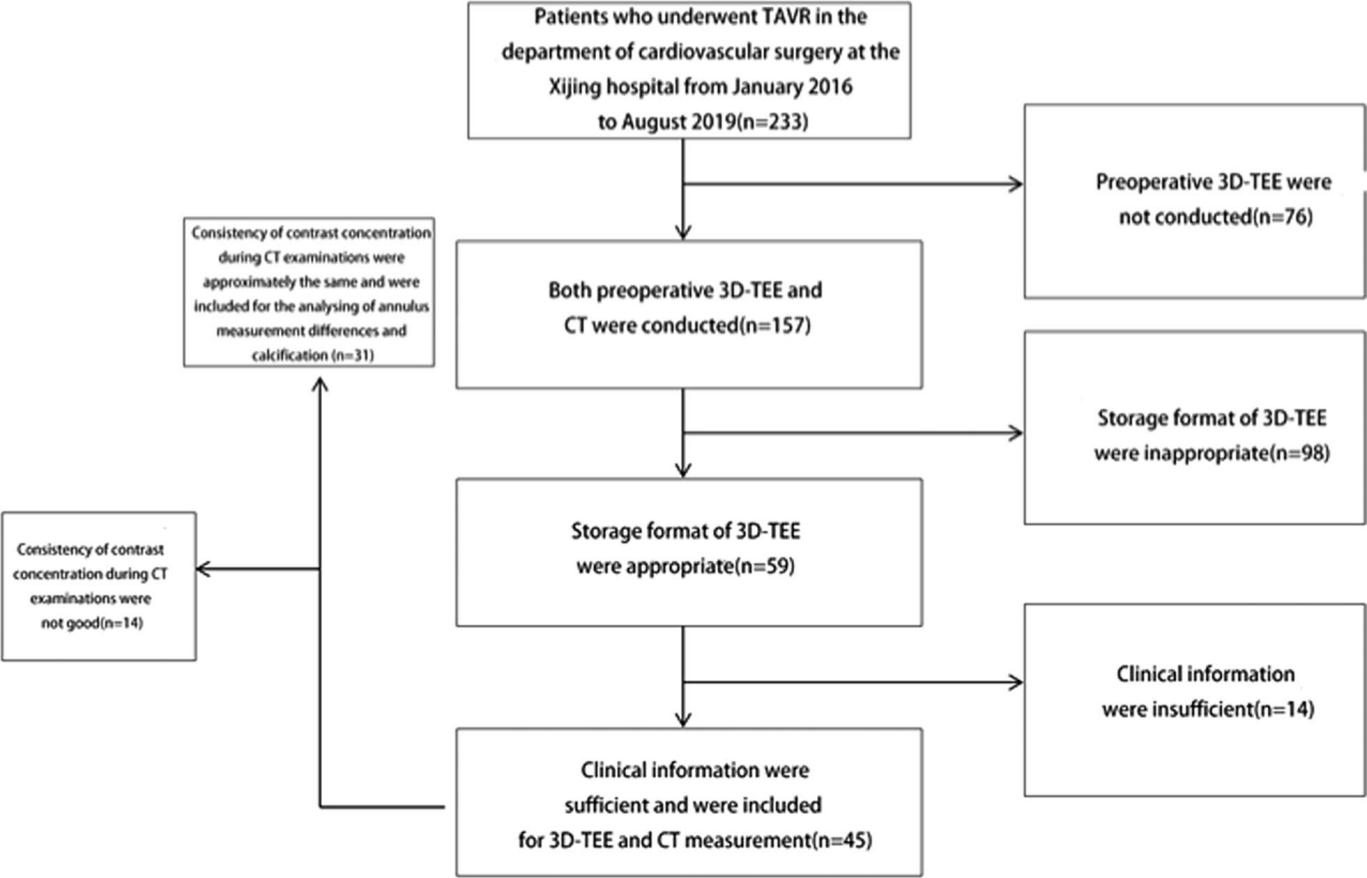
The screening process of patients

We retrospectively collected information on 233 patients who underwent TAVR from January 2016 to August 2019 in the department of cardiovascular surgery, Xijing Hospital. A total of 45 patients whose preoperative CT and intraoperative 3D-TEE data were available and attainable were included in our study. Other patients were excluded because of insufficient image acquisition or inappropriate storage format of 3D-TEE data. The screening process was shown as Fig. 2.

### Image acquisition and evaluation

Preoperative CT images were stored in the Digital Imaging and Communications in Medicine format, which is the international standard storage format for medical images and related information. After the data were imported into Materialise Mimics 21.0 (Leuven, Belgium), a set was composed. In addition to the original axial plane, the software automatically reconstructed the sagittal and coronal planes. To make sure the three lowest points of the aortic sinus simultaneously appeared in the axial plane, we adjusted the location of the planes and obtained the virtual annulus plane. After manually delineating the contour of the annulus, the information we needed, such as the area-derived diameter of the annulus (annulus-Da), the perimeter-derived diameter of the annulus (annulus-Dp), and the mean diameter^1^ of the annulus (annulus-mean), could be collected. Using the same method, other measurements such as the perimeter-derived diameter of the left ventricular outflow tract (LVOT-Dp), the perimeter-derived diameter of the sinotubular junction, and the diameter of the sinus of Valsalva (SOV) could also be obtained.

The 3D-TEE data were obtained immediately before the operation using the Philips Medical ultrasound system (iE33) and probe (X7-2t) (Philips Medical Systems, Amsterdam, the Netherlands). Images were also stored in the Digital Imaging and Communications in Medicine format, imported into QLAB software (Philips Medical Systems), and viewed in the 3DQ mode. We chose images in the proper phase and adjusted the three planes perpendicular to the aortic root so that the cross-section just passed through the aortic lobe to attach to the plane at the lowest point, which is called the aortic ring plane. The area of the aortic annulus was measured on this plane, and the mean value of the annulus diameter was calculated using the formula for the area of a circle (A = πR^2^). Other data, such as the STJ and LVOT, were obtained by moving the valve ring plane up and down. The severity of valve calcification was evaluated using HU450 and HU850, which were defined as the volume of the part whose CT value is more than 450 or 850 HU; these values are commonly used in preoperative evaluation of patients having TAVR. These two factors can be evaluated using Materialise Mimics software.

### Statistical analyses

The Kolmogorov–Smirnov test was performed. Categorical variables are presented as number (percentage). If a normal distribution is confirmed, continuous data are presented as the mean ± SD. A paired two-sided Student *t*-test, Pearson correlation coefficients, and linear regression were used to compare measurements between the two methods. For those measurements that were not normally distributed, continuous data are presented as the median (P25, P75). The differences between the two methods were evaluated using the Wilcoxon signed-rank test. The correlations were calculated using the Spearman rank test. A normal transformation was used if needed. Bland–Altman plots were used to calculate the concordance between 3D-TEE and CT. To explore the influence of calcification on the differences between the two methods, a Spearman rank test was conducted with the Linear regression analysis obtained. Statistical significance was set at *P* < 0.05. Data analyses were conducted with SPSS 23 (SPSS Inc., Chicago, IL, USA).

## Results

### Patients

**Fig. 3:**
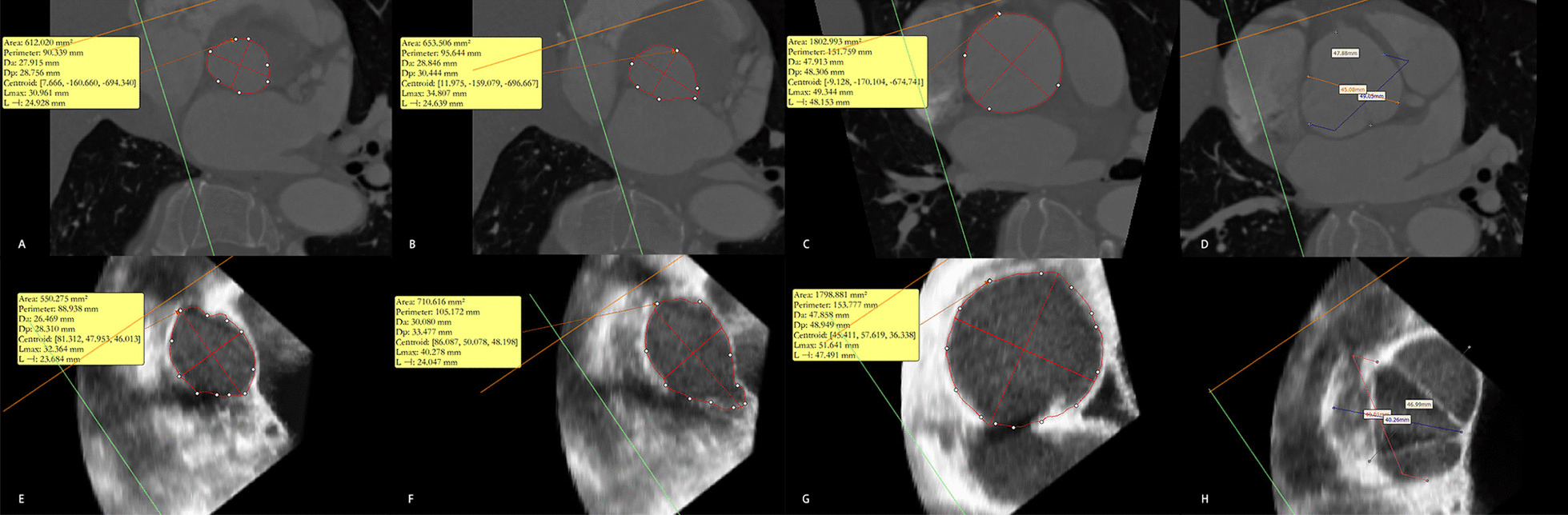
Transcatheter aortic valve replacement steps at the Xijing hospital. **A**–**D** Insertion of a J-valve via a transapical approach. **E**–**H** Inserting a Venus-A valve via a transfemoral approach

The patients were 67.2 ± 7.5 years old; 35 (77.8%) were men. Using preoperative transthoracic echocardiography, 10 (22.2%) were diagnosed with pure AS, 17 (37.8%) with pure AR, and 18 (40.0%) with AS with regurgitation. A total of 44 of 45 patients received TAVR except for 1 who was not operated on because of a severely narrowed LVOT. We used a transapical approach with the J-valve (Jie Cheng Medical ltd., Su Zhou, China) in 22 (48.9%) of them. For the others, we used the transfemoral approach with the Venus-A valve (QI Ming Medtech Ltd, Hang Zhou, China) (Fig. 3). One (2.2%) patient was converted to a thoracotomy because of valve migration; 1 (2.2%) patient with unstable blood pressure was placed on extracorporeal membrane oxygenation. The other 42 cases were successfully operated on (Table 1).

**Table 1:** Demographic and Clinical Baseline Characteristics

Age (years)	67.2 ± 7.5
Male	35 (77.8%)
STS-PROM (%)	4.97 (3.35, 5.61)
Logistic EuroSCORE II score (%)	11.30 (3.48, 13.67)
Hematocrit	0.40 (0.38, 0.45)
WBC (× 10^9^)	7.58 (5.48, 9.32)
Platelet(× 10^6^)	188.58 (151.50, 214.00)
Creatinine (µmol/L)	91.64 (69.00, 104.00)
Left ventricular ejection fraction (%)	44.36 (34.50, 54.50)
Hypertension	24 (53.3%)
Cerebrovascular Disease	16 (35.5%)
Diabetes	12 (26.7%)
Chronic Lung Disease	27 (60.0%)
Classification-NYHA	43 (95.6%)
Atrial fibrillation	11 (24.4%)
Coronary artery disease	13 (28.9%)
Aortic stenosis	10 (22.2%)
Aortic regurgitation	17 (37.8%)
Aortic stenosis with regurgitation	18 (40.0%)
Transapical	22 (48.9%)
Transfemoral	22 (48.9%)
Turn to ECMO	1 (2.2%)
Trans to surgery	1 (2.2%)

### Annulus and left ventricular outflow tract measurements

**Fig. 4:**
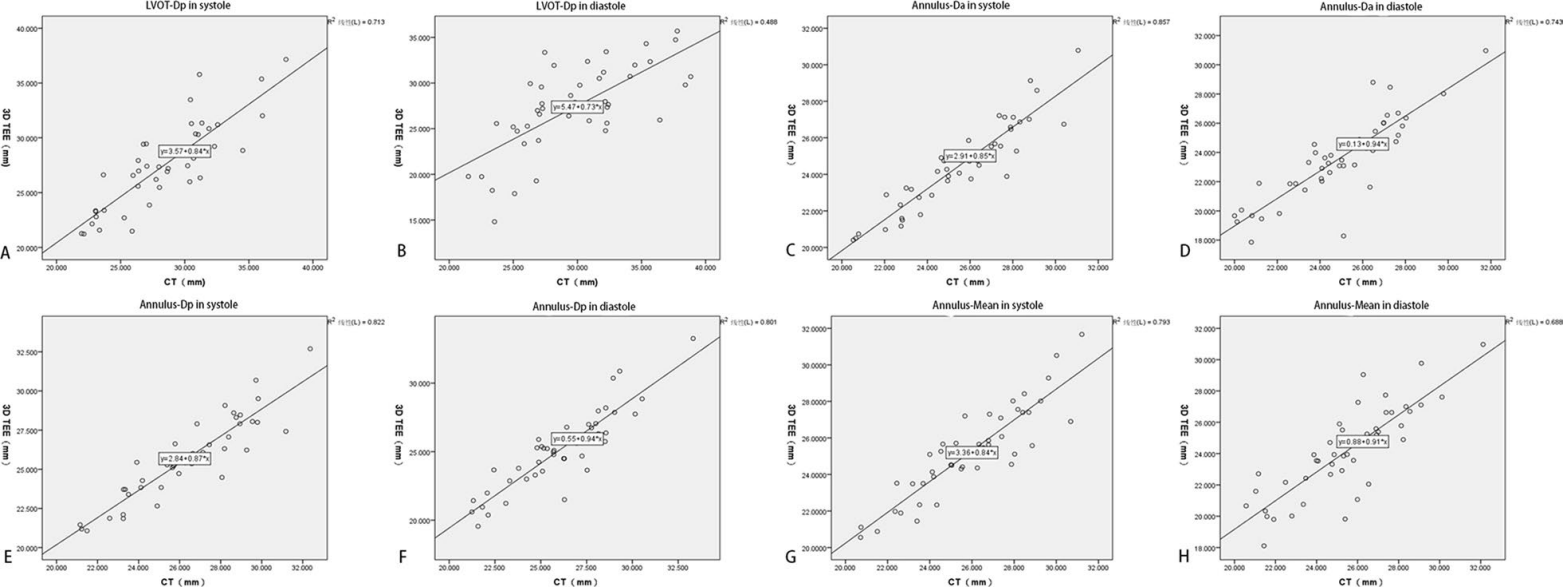
Linear regression showing high correlations between computed tomography and 3-dimensional-transesophageal echocardiography measurements of the left ventricular outflow tract (LVOT) and the annulus. **A** LVOT-perimeter-derived in systole. **B** LVOT-perimeter-derived in diastole. **C** Annulus-area-derived in systole. **D** Annulus-area-derived in diastole. **E** Annulus-perimeter-derived in systole. **F** Annulus-perimeter-derived in diastole. **G** Annulus-mean in systole. **H** Annulus-mean in diastole

**Fig. 5:**
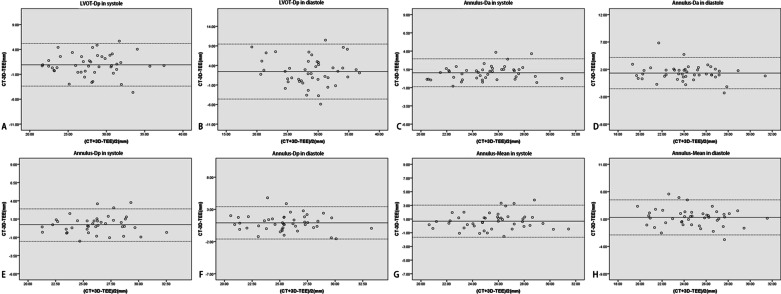
Bland–Altman plot showing good agreement between computed tomography and 3-dimensional transesophageal echocardiography measurements of the left ventricular outflow tract (LVOT) and annulus. **A** LVOT-perimeter-derived in systole. **B** LVOT-perimeter-derived in diastole. **C** Annulus-area-derived in systole. **D** Annulus-area-derived in diastole. **E** Annulus-perimeter-derived in systole. **F** Annulus-perimeter-dreived in diastole. **G** Annulus-mean in systole. **H** Annulus-mean in diastole

All of the CT and 3D-TEE images for the 45 patients were suitable for annulus and LVOT evaluation, and normal distributions were confirmed. Compared to the CT references, measurements derived from 3D-TEE data obviously underestimated the diameter of the aortic annulus and the LVOT: annulus-Da in systole ([24.52 ± 2.36] vs. [25.55 ± 2.59] mm; *P* < 0.01); annulus-Dp in systole ([25.69 ± 2.56] vs. [26.36 ± 2.67] mm; *P* < 0.01); annulus-mean in systole ([25.24 ± 2.47] vs. [25.93 ± 2.61] mm; *P* < 0.01); LVOT-Dp in systole ([27.57 ± 3.91] vs. [28.48 ± 3.92] mm; *P* < 0.01); annulus-Da in diastole ([23.54 ± 2.86] vs. [24.86 ± 2.61] mm; *P* < 0.01); annulus-Dp in diastole ([25.11 ± 2.85] vs. [26.02 ± 2.70] mm; *P* < 0.01); annulus-mean in diastole ([24.14 ± 2.90] vs. [25.44 ± 2.63] mm; *P* < 0.01); LVOT-Dp in diastole ([27.37 ± 4.73] vs. [29.79 ± 4.50] mm; *P* < 0.01) (Table 2). The linear regression and correlation coefficients (range 0.70–0.95) showed that the two measurements were closely related to each other (*P* < 0.01) (Fig. 4); the Bland–Altman analysis showed they were in good agreement (Fig. 5).

**Table 2:** Comparison and correlation analysis of 3D-TEE and CT measurement of annulus and LVOT

Measurement	Sample size	3D-TEE	CT	CT-TEE	*P*	Correlation	
						R	*P*
*Systole*							
Annulus-Da	45	24.52 ± 2.36	25.55 ± 2.59	1.03 ± 0.98	< 0.01	0.93	< 0.01
Annulus-Dp	45	25.69 ± 2.56	26.36 ± 2.67	0.67 ± 1.13	< 0.01	0.91	< 0.01
Annulus-Mean	45	25.24 ± 2.47	25.93 ± 2.61	0.69 ± 1.20	< 0.01	0.89	< 0.01
LVOT-Dp	45	27.57 ± 3.91	28.48 ± 3.92	0.91 ± 2.19	< 0.01	0.84	< 0.01
*Diastole*							
Annulus-Da	45	23.54 ± 2.86	24.86 ± 2.61	1.32 ± 1.46	< 0.01	0.86	< 0.01
Annulus-Dp	45	25.11 ± 2.85	26.02 ± 2.70	0.91 ± 1.28	< 0.01	0.90	< 0.01
Annulus-Mean	45	24.14 ± 2.90	25.44 ± 2.63	1.30 ± 1.63	< 0.01	0.83	< 0.01
LVOT-Dp	45	27.37 ± 4.73	29.79 ± 4.50	2.42 ± 3.59	< 0.01	0.70	< 0.01

### SOV and STJ measurements

**Fig. 6:**
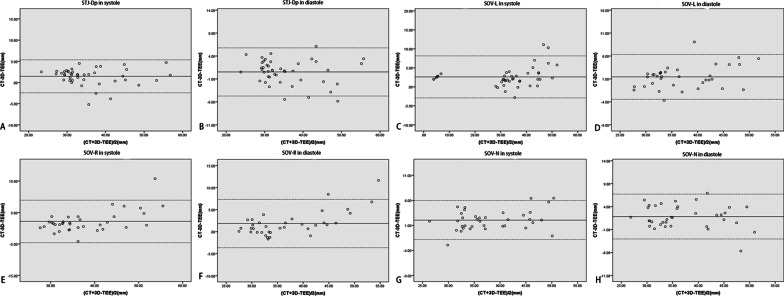
Bland–Altman plot showing good agreement between computed tomography and 3-dimensional transesophageal echocardiography measurements of the sinotubular junction (STJ) and the sinus of Valsalva (SOV). **A** STJ-perimeter-derived in systole. **B** STJ-perimeter-derived in diastole. **C** SOV-left coronary in systole. **D** SOV-left coronary in diastole. **E** SOV-right coronary in systole. **F** SOV-right coronary in diastole. **G** SOV-noncoronary in systole. **H** SOV-noncoronary in diastole

**Fig. 7:**
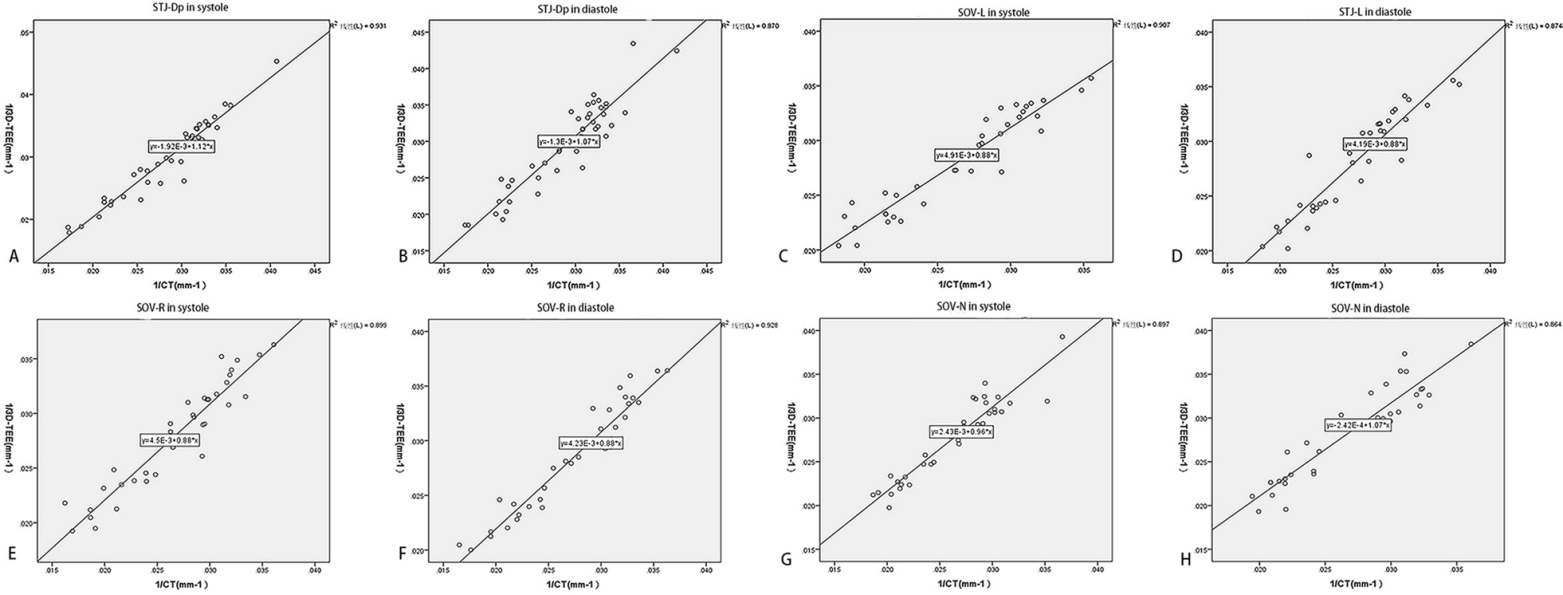
After the reciprocal transformation of the computed tomography and 3-dimensional transesophageal echocardiography measurements of the sinotubular junction (STJ) and the sinus of Valsalva (SOV), all the data approximately obeyed the normal distribution. Linear regression shows a high correlation between the two imaging modalities. **A** STJ-perimeter-derived in systole. **B** STJ-perimeter-derived in diastole. **C** SOV-left coronary in systole. **D** SOV-left coronary in diastole. **E** SOV-right coronary in systole. **F** SOV-right coronary in diastole. **G** SOV-noncoronary in systole. **H** SOV-noncoronary in diastole

Nine patients with bicuspid aortic valves were excluded from the SOV measurement because the sample size was too small. Two patients in systole during 3D-TEE and 1 patient in diastole during 3D-TEE did not qualify for an STJ evaluation because the images were insufficient. Some of the data were treated as being abnormally distributed because they did not fit a normal distribution according to the Kolmogorov–Smirnov test. Except for the perimeter-derived diameter of the sinotubular junction in diastole, which did not show a statistical difference (*P* = 0.11) between the two methods, the other measurements demonstrated that the values from 3D-TEE were significantly smaller than those from CT (*P* < 0.01) (Table 3). The correlation coefficients calculated by the Spearman rank test ranged from 0.89 to 0.95 (*P* < 0.01) and showed a tight correlation between the groups (Table 4). The Bland–Altman study showed that the ordinate values were mostly within the 95% consistency limit; the consistency of the two groups was good (Fig. 6). After the reciprocal transformation of the data, all the data approximately obeyed the normal distribution. The transformed data were used for linear regression analysis (Fig. 7).

**Table 3:** Comparison of 3D-TEE and CT measurement of STJ and SOV

Measurement	Sample size	3D-TEE	CT	*P*
*Systole*				
STJ-Dp	43	31.89 (29.00, 38.79)	33.41 (31.23, 39.45)	< 0.01
SOV-L	36	35.63 (31.04, 42.51)	36.28 (32.75, 46.08)	< 0.01
SOV-R	36	34.49 (31.75, 42.03)	35.51 (32.26, 45.66)	< 0.01
SOV-N	36	34.98 (31.73, 42.95)	37.13 (34.05, 45.83)	< 0.01
*Diastole*				
STJ-Dp	44	32.59 (29.51, 40.23)	32.77 (30.77, 39.65)	0.11
SOV-L	36	34.71 (31.43, 41.51)	35.53 (32.37, 43.24)	< 0.01
SOV-R	36	34.09 (30.37, 41.61)	33.87 (31.56, 44.60)	< 0.01
SOV-N	36	33.97 (30.63, 42.48)	35.70 (32.59, 45.08)	< 0.01

*SOV-L* left-coronary sinus of Valsalva, *SOV-R* right-coronary sinus of Valsalva, *SOV-N* non-coronary sinus of Valsalva

**Table 4:** Correlation analysis of 3D-TEE and CT measurement of STJ and SOV

Measurement	Sample	3D-TEE	CT	Correlation		1/TEE*	1/CT*	Correlation	
				R	*P*			R	*P*
*Systole*									
STJ-Dp	43	31.89 (29.00, 38.79)	33.41 (31.23, 39.45)	0.95	< 0.01	0.030 ± 0.006	0.029 ± 0.005	0.97	< 0.01
SOV-L	36	35.63 (31.04, 42.51)	36.28 (32.75, 46.08)	0.93	< 0.01	0.028 ± 0.005	0.026 ± 0.005	0.95	< 0.01
SOV-R	36	34.49 (31.75, 42.03)	35.51 (32.26, 45.66)	0.95	< 0.01	0.028 ± 0.005	0.027 ± 0.005	0.95	< 0.01
SOV-N	36	34.98 (31.73, 42.95)	37.13 (34.05, 45.83)	0.92	< 0.01	0.028 ± 0.005	0.026 ± 0.004	0.95	< 0.01
*Diastole*									
STJ-Dp	44	32.59 (29.51, 40.23)	32.77 (30.77, 39.65)	0.86	< 0.01	0.030 ± 0.006	0.029 ± 0.005	0.93	< 0.01
SOV-L	36	34.71 (31.43, 41.51)	35.53 (32.37, 43.24)	0.9	< 0.01	0.028 ± 0.005	0.027 ± 0.005	0.94	< 0.01
SOV-R	36	34.09 (30.37, 41.61)	33.87 (31.56, 44.60)	0.83	< 0.01	0.029 ± 0.005	0.027 ± 0.005	0.96	< 0.01
SOV-N	36	33.97 (30.63, 42.48)	35.70 (32.59, 45.08)	0.7	< 0.01	0.029 ± 0.005	0.027 ± 0.004	0.93	< 0.01

*SOV-L* left-coronary sinus of Valsalva, *SOV-R* right-coronary sinus of Valsalva, *SOV-N* non-coronary sinus of Valsalva

^*^The data approximate obey to normal distribution after inverse transformation, so they were used for linear regression analysis

### Annulus measurement differences and calcification

The accuracy of the evaluation of HU450 and HU850 depended on the consistency of contrast concentration during CT examinations in different patients. Thirty-one of the 45 patients whose contrast concentrations were approximately the same were included in the study.

There were no statistical correlations between annulus measurement differences and HU450 (*P* = 0.18–0.99) or HU850 (*P* = 0.41–0.83) (Table 5). Similarly, linear regression analysis indicated that the value for calcification as a factor to predict the accuracy of 3D-TEE evaluation was limited. (*R* = 0.01–0.24, *P* = 0.20–0.97) (Table 6).

**Table 5:** Correlation analysis of aortic root calcification and accuracy of 3D-TEE measurement (CT measurement as the golden standard)

Measurement	Sample	|3D-TEE-CT|	HU450	HU850	Correlation (*P*)	
					HU450	HU850
*Systole*						
Annulus-Da	31	1.06 (0.37, 1.61)			0.18	0.41
Annulus-Dp	31	0.87 (0.45, 1.53)			0.99	0.80
Annulus-Mean	31	1.01 (0.47, 1.30)			0.91	0.70
*Diastole*			431.62 (6.61, 1109.81)	95.80 (0.56, 479.92)		
Annulus-Da	31	1.77 (0.97, 2.28)			0.96	0.83
Annulus-Dp	31	1.22 (0.48, 1.84)			0.76	0.75
Annulus-Mean	31	1.72 (0.92, 2.35)			0.75	0.80

**Table 6:** Linear regression analysis of aortic root calcification and accuracy of 3D-TEE measurement (CT measurement as the golden standard)

Measurement	Sample	|3D-TEE-CT|	HU450			HU850		
				R	*P*		R	*P*
*PPSystole*								
Annulus-Da	31	1.06 (0.37, 1.61)		0.24	0.20		0.20	0.28
Annulus-Dp	31	0.87 (0.45, 1.53)		0.15	0.44		0.09	0.63
Annulus-Mean	31	1.01 (0.47, 1.30)		0.20	0.28		0.19	0.30
*Diastole*			431.62 (6.61, 1109.81)			95.80 (0.56, 479.92)		
Annulus-Da	31	1.77 (0.97, 2.28)		0.02	0.91		0.01	0.97
Annulus-Dp	31	1.22 (0.48, 1.84)		0.05	0.80		0.02	0.92
Annulus-Mean	31	1.72 (0.92, 2.35)		0.04	0.82		0.05	0.79

## Discussion

CT is the standard image modality for preoperative evaluation of patients being considered for TAVR [11]. Its sharp resolution of vascular and nonvascular tissues makes the measurement results more accurate and reliable. However, for patients with renal insufficiency, contrast medium allergy, or other special conditions, the application of CT is often limited [12]. Some patients with unstable hemodynamics who needed emergency procedures could not have a CT scan. 3D-TEE has been regarded as an alternative image evaluation resource because it causes no harm to the human body. However, high quality 3D-TEE imaging has very strict requirements for the control of respiration and heart rate and is vulnerable to the interference of calcification and other factors. Based on 3D-TEE data, there is no unified standard measurement method. Current studies in the literature generally show that 3D-TEE based on an evaluation of the aortic root underestimates the measurement results and leads to a higher incidence of complications [13]. However, there is no convincing research on the causes of these differences. We found that the published studies all used different software and methods to evaluate the CT and 3D-TEE data. The inconsistency of this evaluation tool and method may be one of the reasons for the poor accuracy of a 3D-TEE evaluation. In addition, older patients undergoing a TAVR operation often have severe calcification, which is an important factor affecting the image quality. Its impact on 3D-TEE is often more serious than its impact on CT. We suspect that the existence of calcification may be another factor that leads to the significant difference between 3D-TEE and CT evaluations, thus affecting the accuracy of the 3D-TEE evaluation. To verify whether the differences in measurements between 3D-TEE and CT are caused by the inconsistencies in the software and methods, Materialise Mimics software, which can be used to analyze CT images and 3D-TEE data, was used to avoid the possible errors.

Preoperative evaluation of CT scans based on the multiplane reconstruction function of the measurement software, such as 3mensio (Pie Medical Imaging BV, Maastricht, the Netherlands) is considered the gold standard for patients undergoing TAVR. The obvious contrast between the lumen and the myocardium/vascular wall enables its significant performance in distinguishing vascular and nonvascular structures. However, the use of contrast and the exposure to ionizing radiation limit its application in patients who are allergic to contrast agents or who have renal insufficiency. In some patients, such as patients requiring emergency surgery, patients with atrial fibrillation, and patients with renal failure, it is difficult to perform a CT examination. In these cases, we usually choose 3D-TEE as the preoperative examination method; it can also serve as a real-time monitoring method during the operation. 3D-TEE was expected to be an alternative method for these patients. In contrast to CT, there is no mature or uniform TEE-based preoperative evaluation method. 2D-TEE was the initial resource. However, because the structures being measured were usually oval instead of perfectly round, the measurements tended to severely underestimate the results. As 3D-TEE became more popular, it replaced 2D-TEE and was widely used in the preoperative evaluation of patients scheduled for TAVR. Arnold [14] used QLAB (Philips Medical Systems) to measure the aortic annulus and LVOT by the same multiplane reconstruction method as CT. Omar [15] evaluated the aortic root by the off-label use of the mitral valve measurement software Mitral Valve Quantification (Philips Medical Systems). A semi-automated software specifically designed for the aortic root was used by Mediratta and his colleagues [16]. Two automated software systems, Aortic Valve Navigator (Philips Medical Systems) and eSieValvesTM (Siemens Healthineers, Erlangen, Germany), were used by Edgard [17] and Nahoko [18], respectively. Despite improvements in software, the foregoing studies generally showed that measurements based on 3D-TEE were still significantly smaller than those based on CT. We hypothesized that the inconsistencies between the methods and the software might be important factors contributing to the measurement differences. Therefore, we used the same Materialise Mimics software to evaluate CT and 3D-TEE images to eliminate the possible error. However, the final results were not as we expected. The difference was not significantly reduced. Nevertheless, good correlation and agreement between them still enabled us to use 3D-TEE as preoperative imaging data in patients having TAVR who had contraindications for a CT examination. Figure 2 shows the regression equation between CT and 3D-TEE measurements regarding the annulus and the LVOT.

Severe calcification significantly worsens the quality of 3D-TEE images because of the artifact it generates, thus, in theory, impeding the measurement and reducing its accuracy. Nevertheless, our results failed to provide the expected conclusion. Calcification did not show an obvious correlation with the differences between the 3D-TEE and CT measurements of annulus diameters. The value of HU450 and HU850 in predicting the discrepancy was also limited from the analysis of the ROC curve. In fact, we found that calcification was not always a negative factor: Its highlighting signal can sometimes help to distinguish the valve leaflet and to locate the annulus, which may partly account for the results.

What is new about our research is not only the fact that we used the same software and method for the 3D-TEE and CT measurements, but also that we expanded the patient sample and measurement parameters. Due to the lack of calcification, which was essential to anchor the bioprosthetic valves in the early cases, patients with pure AR were once considered to be contraindicated for TAVR [19]. In recent years, CoreValve (Medtronic Inc., Minneapolis, MN, USA) [20] and SAPIEN XT (Edwards Lifesciences Inc., Irvine, CA, USA) [21], with the help of special designs and new valves dedicated for use in patients with AR, such as JenaValve (JenaValve Technology GmbH, Munich, Germany) [22], J-valve (Jie Cheng Medical ltd., Su Zhou, China) [23], and the ACURATE series (Symetis SA, Ecublens, Switzerland) [24, 25], had started to be used for the treatment of these patients. To our knowledge, this study is one of the first to include patients with AR. The preoperative evaluation of patients scheduled for TAVR mainly included measurements of the diameters of the aortic annulus, LVOT, SOV, STJ, and the ascending aorta and assessment of coronary artery height, left ventricular aortic angle, valve type and calcification, optimal angiographic projection angle, and peripheral vascular anatomy. Each parameter plays an important role in developing strategy. Accurate acquisition of the diameters of the annulus and the other structures can help the surgical team to choose the most appropriate bioprosthetic valve and the best deployment area to prevent complications such as valve migration and paravalvular leak. Coronary height and SOV volume are critical indicators needed to judge the risk of myocardial infarction. Left ventricular aorta angle and peripheral blood vessel evaluation are essential for selecting a better approach, and the optimal angiographic projection angle enables the surgical team to achieve the best surgical vision. In contrast to previous studies, which focused mainly on the annulus, we also compared the measurements of the LVOT, the SOV, and the STJ. The results showed that 3D-TEE scans can be used as an alternative for their evaluation.

How to reach the most accurate preoperative evaluation has always been the issue of greatest concern. In addition to making full use of image resources, 3D printing technology provides new approaches. Through in vitro reconstruction of a patient-specific aortic root model and precise presentation of the anatomical structure, 3D printing models have played a significant role in the preoperative evaluation of patients being considered for TAVR. With the development of 3D printing materials, in combination with finite element analysis, we look forward to being able to select the best operative strategy and to forecast operative results by computer simulation before the operation in order to further improve our success rate and reduce the risk of complications.

### Study limitations

This study was retrospective and involved a single center. The implanted J-valves and the Venus-A valve are different from prosthetic valves used in other countries. In addition, because of the small sample size, some of the STJ and SOV data did not follow a normal distribution and therefore could not be used for regression equations and consistency analysis, which may have weakened the representativeness and persuasiveness of the results. We have not attempted to compare 3D-TEE and CT findings with the findings obtained with other methods to make clinical decisions. We will add a rigorous comparison of more imaging methods in future research.

## Conclusion

High correlation and good agreement between the two sets of measurements enable 3D-TEE to be an appropriate alternative for patients requiring TAVR who cannot undergo a CT scan as the image resource for preoperative individualized decision making. Differences between 3D-TEE and CT measurements still exist even when one uses the same software and the same method. Other factors that may affect the difference need to be explored.

## Data Availability

The datasets used and/or analysed during the current study are available from the corresponding author on reasonable request.

## Abbreviations

3D-TEE: Three-dimensional transesophageal echocardiography
annulus-Da: Area-derived diameter of the annulus
annulus-Dp: Perimeter-derived diameter of annulus
annulus-mean: Mean diameter of annulus
AR: Aortic regurgitation
AS: Aortic stenosis
CT: Computed tomography
LVOT-Dp: Perimeter-derived diameter of the left ventricular outflow tract
SOV: Sinus of Valsalva
TAVR: Transcatheter aortic valve replacement

## Footnotes

1 Mean diameter = $$\frac{{{\text{maximum diameter}} + {\text{minimal diameter}}}}{2}.$$
